# A Curiosity in Obstetric Physiology

**Published:** 1843-08

**Authors:** John H. Griscom

**Affiliations:** One of the Physicians of the New York Hospital


*A Curiosity in Obstetric Physiology.*—By John H. Griscom, M.D., one of the Physicians of the New York Hospital.—In the month of October, 1841, I was consulted by Mrs. R. M. H., for a hæmorrhagic discharge from the vagina, she being pregnant, as she believed, about seven months. This discharge had existed about five months, mostly every day, but sometimes ceasing for a while; but latterly it had become more steady. She described it as apparently not pure blood, but a mixture of blood and water, or very thin blood, of a bright color, and never coagulated. She had felt the motions of the fœtus with considerable distinctness, until within a month, since when they had diminished in strength and frequency, and for the last few days had totally ceased. Her general health (except some muscular pains, and some difficulty in moving about actively), and her appetite, were excellent. She was bled on the 16th, the blood being unusually black. The operation enlivened her much, and she thought she felt some movement of the fœtus, but too indistinct to be certain of it. An examination of the abdomen, in the sitting posture, developed a general enlargement which was quite soft, with none of the uniform and peculiar firmness of the healthy impregnated uterus of that period. Midway between the umbilicus and pubis, the fingers could be pressed back nearly to the prominence of the sacrum. When this was done, there were distinct traces of a tumor, occupying the right iliac fossa, of an elongated form, and very tender to the touch, and a smaller tumor at the left side of the median line. The upper edges of both were distinctly perceived by the hand when pressed gently between them. There was no requirement for any interference until the 24th, when the husband came to me and said he believed she was in labor. I found her sitting up, and having what seemed very much like labor pains, every few minutes. These ceased in a few hours, but recurred slightly the following day. On the 26th she went to bed, complaining of severe pain in the abdomen generally, greatly increasing at short intervals, but not subsiding entirely at the intermissions.

An examination, per vaginam, was now made, and a firm round tumor was perceived, giving the distinct impression of a fœtal head felt through the parietes of the uterus. It was movable, as is ordinary in pregnancy, and motion was communicable through it to the abdomen.

The abdomen now became very tender to the touch, especially over the right tumor; the pulse rose to one hundred and thirty, and the tongue became coated; and though both tongue and skin remained most, the tenderness of the abdomen soon became so excessive, that peritonitis appeared severely developed. The hæmorrhage from the vagina now disappeared, and did not recur. Active antiphlogistic treatment was adopted; and each successive effort produced a subsidence of the symptoms, but for a few minutes only. The flame would suddenly light up again, after a short period of ease to the patient, and of hope to the attendants, and burn with increased intensity, until, at the end of the week, she sank calmly in death.

When confined to the bed, Mrs. H. first informed me that she had *always* doubted her being pregnant, though she had had many of the usual symptoms, such as enlargement of the mammæ. with exudation of their milk, motions of fœtus, etc.; but the latter were often so obscure, as to lead her to hesitate between them and borborygmi.

*Autopsia* in company with Drs. Boyd, Swett, and S. T. Smith.—The interior abdomen presented the usual appearances of inflammation of the peritoneum and intestines, with great vascular injection of the omentum, and loss of its substance; and among the folds of the bowels and through the cellular tissue, there was a larger tumor, of a blue color, and towards the left was the uterus, about the size of that organ in the second month of pregnancy. Extensive recent adhesions existed between the bowels and the tumor, and in separating them, several ulcerated holes were discovered in the membrane covering the tumor.

The tumor was found to dip down into the pelvic cavity, and was subsequently observed to occupy its entire extent. The contents of the pelvis were removed and laid upon a platter for closer inspection. The uterus was first opened longitudinally; it was very healthy looking, except in size, and was entirely void of anything like a fœtus. It contained a small quantity of mucus, and its inner surface was copiously dotted with red points. The left fallopian tube and ovary were sound, as was also the right tube. The latter was traversed with some difficulty by means of bristles, up to the fimbriated extremities, which terminated against the tumor, the latter being in fact the right ovarium, developed to the size of a large cocoa nut. This was opened longitudinally, and immediately there was brought to view a perfectly formed fœtus, of about six months, placenta and all complete. The child appeared to have been dead a considerable time; it was very soft, and the placenta was partially converted into purulent looking matter, similar to that found in the peritoneal cavity. It had escaped through the openings before noticed. The ovarium, as now seen, consisted of a large sac, of a mingled muscular and membranous texture, about as thick as a silver dollar, and was irregularly divided into three or four large cells by membranous partitions. The fœtal head presented towards the vagina.—*N. Y. Journ. of Med.*

